# Relationship Between Epstein–Barr Virus LMP-1 and BRAF-V600E Mutant Protein Expression in Ameloblastoma

**DOI:** 10.3390/dj14090556

**Published:** 2026-09-02

**Authors:** Kenko Okamoto, Michiko Nishimura, Yuji Miyazaki, Miyako Hoshino, Shinnichi Sakamoto, Miki Haruyama, Fumio Ide, Nobuharu Yamamoto, Kentaro Kikuchi

**Affiliations:** 1 Division of Pathology, Department of Diagnostic and Therapeutic Sciences, Meikai University School of Dentistry, Saitama 350-0283, Japan; nishimu@dent.meikai.ac.jp (M.N.); miyako-h@dent.meikai.ac.jp (M.H.); sakashin@dent.meikai.ac.jp (S.S.); idef@dent.meikai.ac.jp (F.I.); k-kikuchi@dent.meikai.ac.jp (K.K.); 2 Division of Oral and Maxillofacial Surgery, Department of Diagnostic and Therapeutic Sciences, Meikai University School of Dentistry, Saitama 350-0283, Japan; haruyama@dent.meikai.ac.jp (M.H.); nyamamot@dent.meikai.ac.jp (N.Y.); 3 Division of Basic Biology, Department of Oral Biology and Tissue Engineering, Meikai University School of Dentistry, Saitama 350-0283, Japan; y-miyazaki@dent.meikai.ac.jp

**Keywords:** Epstein–Barr virus, latent membrane protein-1, ameloblastoma, BRAF-V600E mutant protein, γH2AX

## Abstract

**Background**: Epstein–Barr virus (EBV) enters the human body via saliva. EBV-encoded latent membrane protein-1 (LMP-1) activates the mitogen-activated protein kinase (MAPK) pathway. MAPK signaling pathway activation due to the BRAF-V600E mutation is widely known as a major event in the pathogenesis of ameloblastoma. It is unclear whether EBV infects ameloblastoma and is involved in the pathogenesis of ameloblastoma. We investigated the relationship between LMP-1 and BRAF-V600E mutant protein expression in ameloblastoma by immunohistochemistry. **Methods**: We examined a total of 334 samples (ameloblastoma 117 samples, odontogenic keratocyst 127 samples, dentigerous cysts 45 samples, and dental follicle 45 samples). Ephrin type-A receptor 2 (EphA2), BRAF-V600E mutant protein, and γH2AX expression were confirmed by immunohistochemistry. We confirmed that the positive cases of BRAF-V600E mutant protein and γH2AX segregated based on LMP-1 expression: high grade (HG) and low grade (LG). **Results**: EphA2 expression was high in all lesions. The positive rate of BRAF-V600E mutant protein expression was significantly higher in ameloblastoma, and the positive rate of BRAF-V600E mutant protein in LMP-1 HG cases (71.9%) was significantly greater than in LG cases (39.6%) in ameloblastoma. The positive rate of γH2AX expression was significantly higher in ameloblastoma than in the other lesions. The positive rate of BRAF-V600E mutant protein in LMP-1 HG cases (71.9%) was significantly greater than in LG cases (39.6%) in ameloblastoma. **Conclusions**: BRAF-V600E mutant protein and γH2AX expression in ameloblastoma were significantly higher in LMP-1 HG cases than in LG cases.

## 1. Introduction

Epstein–Barr virus was first isolated from cultured cells of Burkitt’s lymphoma by Epstein et al. in 1964 [1]. EBV is shed in saliva, and most EBV infections occur orally in childhood or early adolescence, resulting in a worldwide infection rate of more than 90% in the adult population [2]. Usually, EBV infection is asymptomatic and latent. EBV infects not only B cells, but also T cells, epithelial cells, and smooth muscle cells [3]. It is well known that EBV is associated with many diseases, including epithelial lesions such as nasopharyngeal carcinoma and gastric carcinoma, and non-epithelial lesions such as Burkitt’s lymphoma and Hodgkin’s lymphoma [3]. In the oral cavity, EBV is associated with a variety of diseases, such as squamous cell carcinoma [4]. EBV-encoded latent membrane protein-1 (LMP-1) is a latent EBV gene associated with host B cell immortalization, transformation of rodent fibroblasts, and enhancement of cell motility and invasion, suggesting that it might function as an oncogene [5]. It has been shown that LMP-1 promotes cell transformation, migration, and invasion in nasopharyngeal epithelial cell lines [6]. LMP-1 also mimics the CD40 receptor and activates several cell signaling pathways, such as the mitogen-activated protein kinase (MAPK) pathway and NF-κB ligand independently [7].

Ephrin type-A receptor 2 (EphA2) belongs to the Eph receptor family expressed on the cell membrane, and the functions of EphA2 are cell migration, adhesion, proliferation, and differentiation [8,9]. In the oral cavity of a mouse, Eph receptor expression is reported during the development of the tongue and palate [10]. It is known that EphA2 is an entry receptor for EBV infection of epithelial cells [9,11].

Most benign odontogenic tumors and cysts are slowly invasive. Ameloblastoma (AM) is a locally invasive benign odontogenic tumor arising in the jaw bones [12]. Most cases of AM arise in the posterior mandible region in younger patients. As AM overexpresses dental epithelial markers such as odontogenic ameloblast-associated protein, it is considered to originate from odontogenic epithelium such as the dental lamina [13,14]. Odontogenic keratocyst (OKC) is classified as a developmental odontogenic cyst arising in the mandible and maxilla, associated with mutation of the *PTCH1* gene, which belongs to the sonic hedgehog signaling pathway [12,15].

The Ras/Raf/MEK/ERK pathway is one of the MAPK pathways and functions in cell proliferation, survival, differentiation, motility, and angiogenesis [16]. BRAF mutation has been found in several malignant neoplasms such as malignant melanoma [17]. Additionally, the presence of the *BRAF*-V600E mutation (valine (V) to glutamic acid (E) substitution at codon 600) has been reported in AM [18,19,20].

Most odontogenic lesions occur in the mandibular molar region of young patients. AM is especially frequent and most often arises in the mandibular molar area. Although many genetic mutations diagnostic for odontogenic tumors remain unknown, the BRAF-V600E mutation has been commonly reported in AM [12]. However, the risk factors for such genetic mutations remain unclear. We reported that the staining intensity of LMP-1 in AM is higher than in OKC, dentigerous cyst (DC), and dental follicle (DF) [21]. We used OKC and DC as comparator lesions because they are high frequency, and used DF as a normal odontogenic epithelium. Therefore, we examined the relationship between LMP-1 expression and BRAF-V600E mutant protein in odontogenic lesions.

## 2. Materials and Methods

### 2.1. Tissue Samples

We examined a total of 334 biopsy and surgical specimens (AM 117 samples, OKC 127 samples, DC 45 samples, and DF 45 samples) retrieved from the hospital of Meikai University School of Dentistry from 1973 to 2020. We used whole sections, and too-small sections were excluded. We used the same samples to perform immunohistochemistry (IHC) to detect LMP-1 in the previous study [21]. We used the assessment for staining intensity modified by Kikuchi et al. [4]. The staining intensity was divided into four levels from 0 to 3, and the sum of the staining intensities of the nucleus and cytoplasm was defined as the total intensity. Total intensity of 0–1 was assigned a score of 0, total intensity 2–3 was scored 1, and total intensity 4–6 was scored 2. As an indicator of the final intensity, we considered scores 0 and 1 to be low grade (LG) and score 2 to be high grade (HG). The assessment of LMP-1 was conducted under simultaneous microscopic observation by two researchers (KO, KK). The numbers of LMP-1 LG and HG cases were DF (LG: 33 cases, HG: 12 cases), DC (LG: 30, HG: 15), OKC (LG: 90, HG: 37), and AM (LG: 53, HG: 64). This study was approved by the Research Ethics Committee of Meikai University School of Dentistry (A1910).

### 2.2. Immunohistochemistry

The IHC data for LMP-1 used in the previous study were also used in the present study [21]. IHC was performed to detect the localization of EphA2, BRAF-V600E mutant protein, and γH2AX in the formalin-fixed, paraffin-embedded (FFPE) sections. After deparaffinization, antigen retrieval was performed using Histofine^®^ antigen retrieval solution (Nichirei Biosciences Inc., Tokyo, Japan; pH 9) for BRAF-V600E mutant protein and Histofine^®^ antigen retrieval solution (Nichirei Biosciences Inc., Tokyo, Japan; pH 6) for γH2AX by microwaving for 10 min. After washing, the sections were immersed for 15 min at room temperature in absolute methanol containing 0.3% H_2_O_2_ to block endogenous peroxidase activity and then treated with 2% bovine serum albumin for 15 min to block non-specific reactions. After washing, the sections were incubated with appropriately diluted primary antibody. The primary antibodies were EphA2 Rabbit polyclonal antibody (1:800 dilution, Cat. No. bs-0485R, Bioss, Woburn, MA, USA), BRAF V600E Rabbit Monoclonal antibody (1:200 dilution, clone RM8, Cat. No. 31-1042-00, RevMAb Biosciences, Dobuque Ave, South San Francisco, CA, USA), and gamma H2AX Rabbit Polyclonal antibody (1:100 dilution, Cat. No. IHC-00059, Bethyl Laboratories, Montgomery, TX, USA). The sections for EphA2 and γH2AX were incubated overnight at 4 °C, and those for BRAF-V600 mutant protein were incubated for 30 min at room temperature. After washing, the sections were incubated with Histofine^®^ Simple Stain MAX-PO(R) (Nichirei Biosciences Inc., Tokyo, Japan), an HRP-labeled polymer reagent for rabbit primary antibodies, at room temperature. They were then immersed for 10 min in 0.05% 3,3′-diaminobenzidine tetrahydrochloride in 0.05M Tris-HCl buffer (pH7.6) containing 0.01% H_2_O_2_ and counterstained with Mayer’s hematoxylin. In a preliminary experiment, we confirmed the staining of the malignant melanoma as a positive control of BRAF-V600E mutant protein. Negative controls were prepared by omitting the primary antibody.

### 2.3. Assessment of EphA2, BRAF-V600E Mutant Protein, and γH2AX

Immunohistochemical staining was evaluated by a single observer. Immunohistochemical staining was considered positive when membranous staining for EphA2, cytoplasmic staining for BRAF-V600E mutant protein, and nuclear staining for γH2AX were observed. Reactivity for EphA2, BRAF-V600E mutant protein, and γH2AX was categorized as positive or negative. The staining was evaluated across the entire tissue section, and representative tumor areas were selected based on the overall staining pattern. Nonspecific reactions or no staining were classified as negative.

### 2.4. Statistical Analysis

All statistical analyses were performed with EZR (version 1.53; Saitama Medical Center, Jichi Medical University, Saitama, Japan) [22]. The significance of differences was determined using the χ2 test or Fisher’s exact test, and the Bonferroni method was used for multiple comparisons. The accepted level of significance was *p* < 0.05.

To evaluate the association between LMP-1 total intensity and BRAF-V600E mutant protein or γH2AX positivity, univariable binary logistic regression analysis was performed. Odds ratios (ORs) and 95% confidence intervals (CIs) were calculated.

## 3. Results

### 3.1. Clinical Characteristics of DF, DC, OKC, and AM

We show the clinical characteristics in Table 1.

The average age of patients with AM, OKC, and DC was in the early forties. In AM, the proportion was the highest among teenagers and tended to decrease in subsequent years. In OKC, the proportion was higher among teenagers and those in their 20s compared to other ages. In DC, the proportion was higher among 30s and 40s compared to other ages. The occurrence site of DF, DC, OKC, and AM was more common in the mandible than the maxilla, and more common in the posterior than the anterior.

We show the number of subtypes of ameloblastoma in Table 2. The number of follicular types was 36 cases, and plexiform types were 42 cases.

### 3.2. Detection of Localization of EphA2 in DF, DC, OKC, and AM

**Figure 1:**
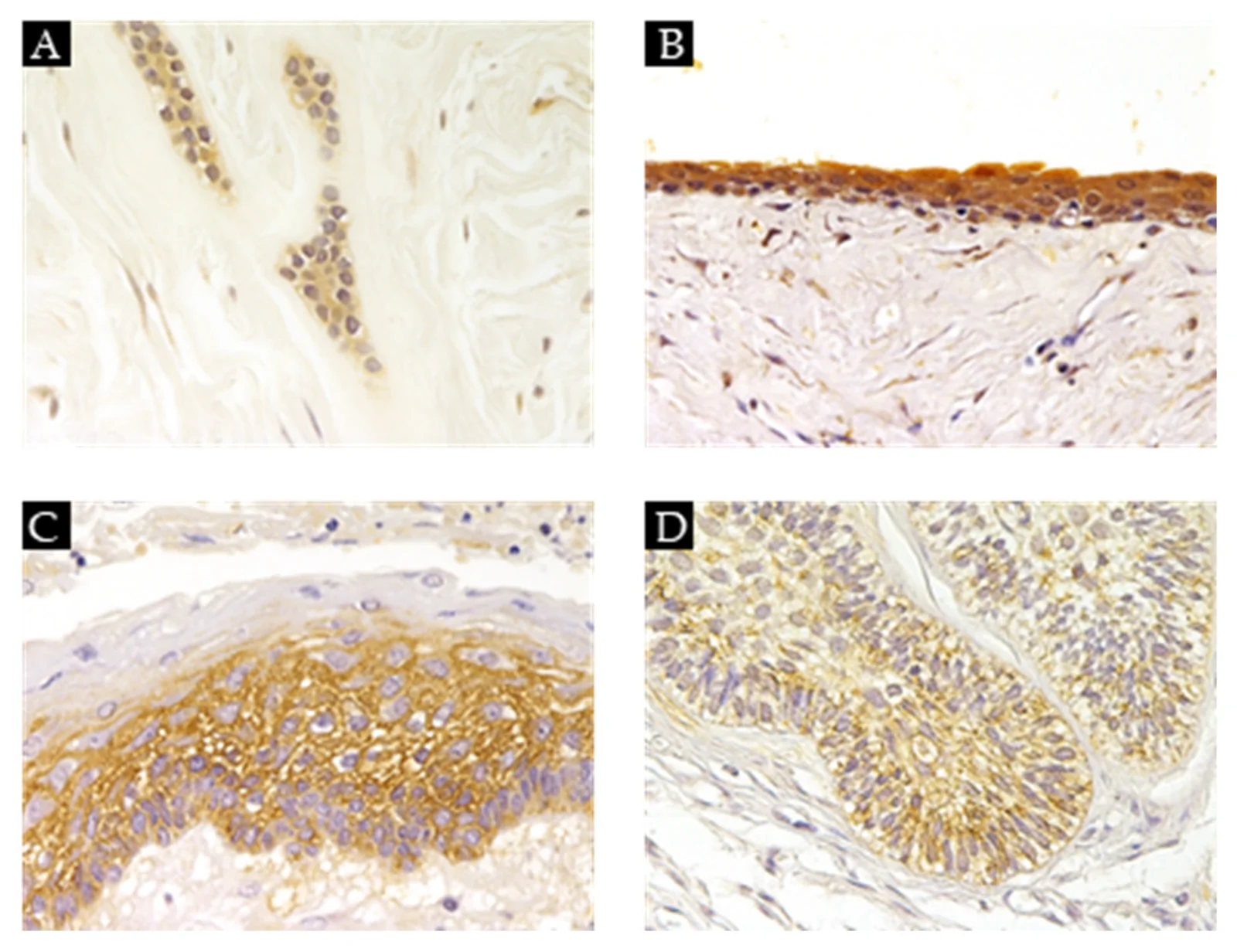
Detection of EphA2 expression by IHC. EphA2 expression was detected in DF (**A**), DC (**B**), OKC (**C**), and AM (**D**). (Original magnification: (**A**–**D**) ×400).

We detected EphA2 expression in the cell membrane in DF, DC, OKC, and AM (Figure 1). The positive rate of EphA2 was 88.9% for DF, 91.1% for DC, 94.5% for OKC, and 90.6% for AM. There were no significant differences among the lesions, but EphA2 expression was high in the epithelium.

### 3.3. Detection of Localization of BRAF-V600E Mutant Protein in DF, DC, OKC, and AM

**Figure 2:**
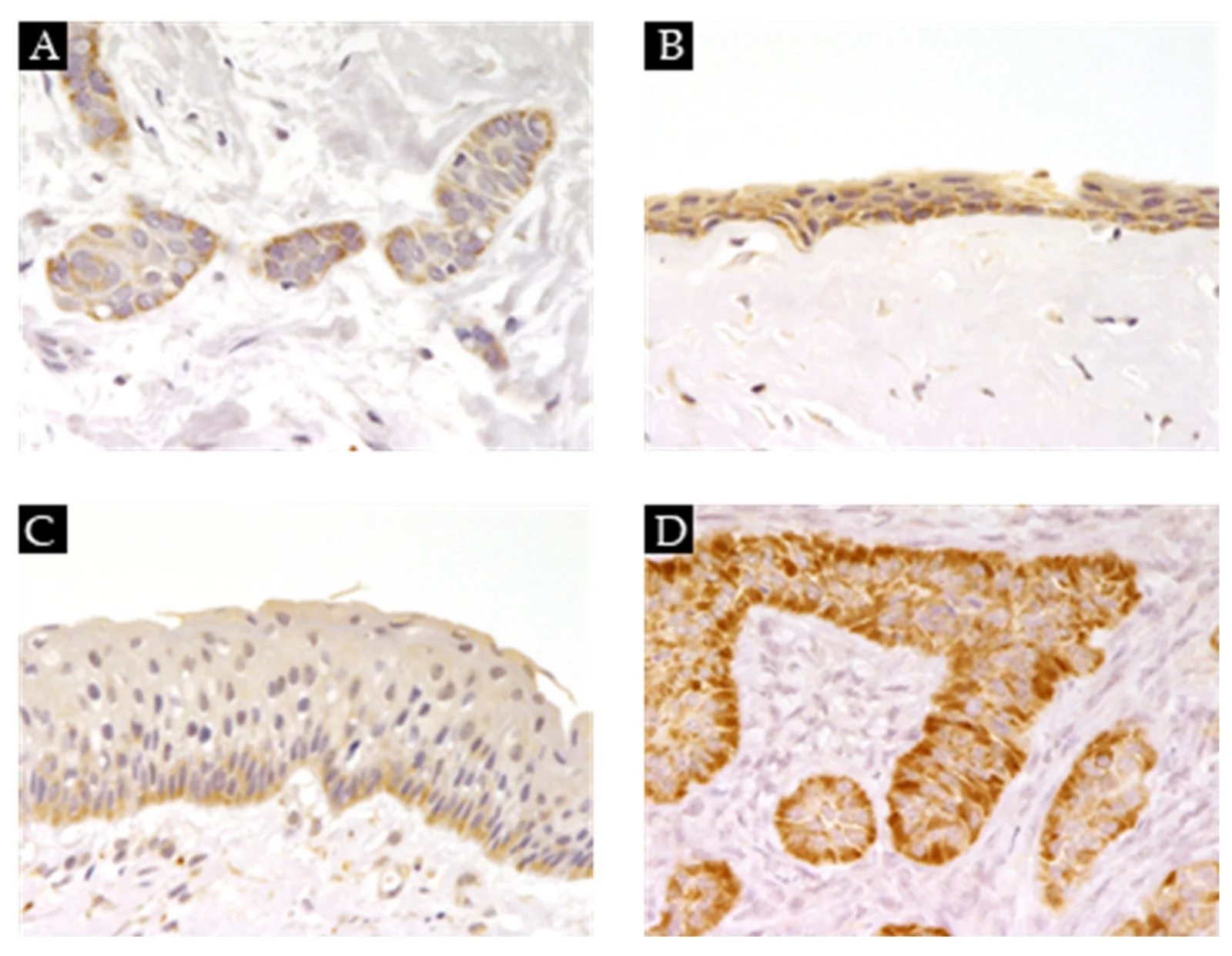
Detection of BRAF-V600E mutant protein expression by IHC. BRAF-V600E mutant protein expression was detected in the cytoplasm of the epithelium of DF (**A**), DC (**B**), OKC (**C**), and AM (**D**). (Original magnification: (**A**–**D**) ×400).

We detected BRAF-V600E mutant protein expression in the cytoplasm in DF, DC, OKC, and AM (Figure 2).

The positive rate of BRAF-V600E mutant protein was 6.7% in DF, 11.1% in DC, 7.1% in OKC, and 54.7% in AM. The positive rate for BRAF-V600E mutant protein expression was significantly higher in AM than in the other lesions by the χ2 test (Table 3).

**Figure 3:**
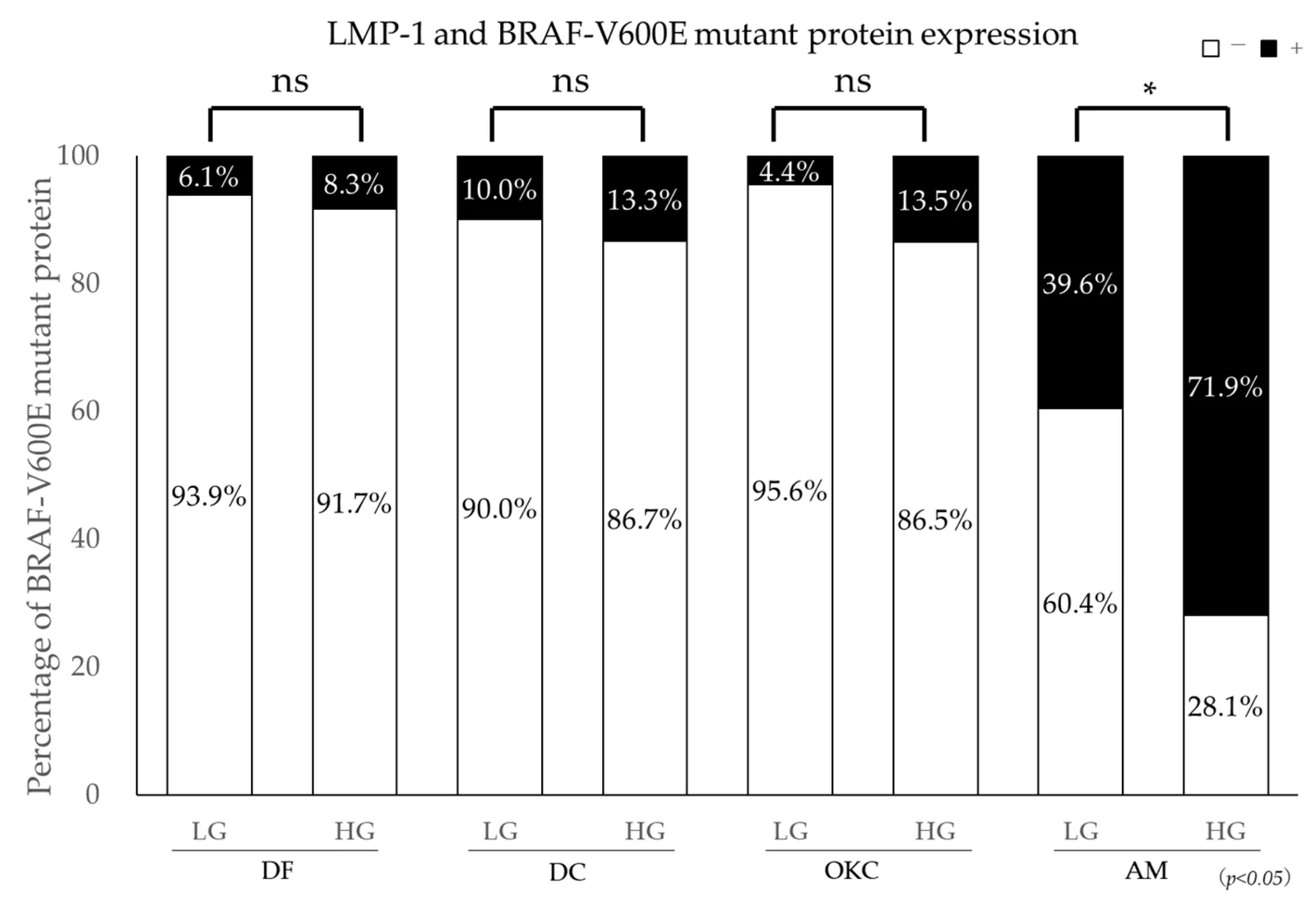
LMP-1 intensity and BRAF-V600E mutant protein expression. The positive rate of BRAF-V600E mutant protein in LMP-1 HG cases (71.9%) was significantly greater than in LG cases (39.6%) in AM. *p*-values were examined by Fisher’s exact test (* *p* < 0.05). ns: not significant.

Regarding the histopathological type of AM, BRAF-V600E mutant protein expression was 63.9% (n = 23/36) in the follicular type and 54.8% (n = 23/42) in the plexiform type, so there was no significant difference between BRAF-V600E mutant protein expression and the follicular type or plexiform type. We investigated the proportion of BRAF-V600E mutant protein in LMP-1 HG and LG cases (Figure 3). In AM, BRAF-V600E mutant protein expression of LMP-1 HG was significantly higher than that of LG (*p* < 0.05).

### 3.4. Detection of Localization of γH2AX in DF, DC, OKC, and AM

**Figure 4:**
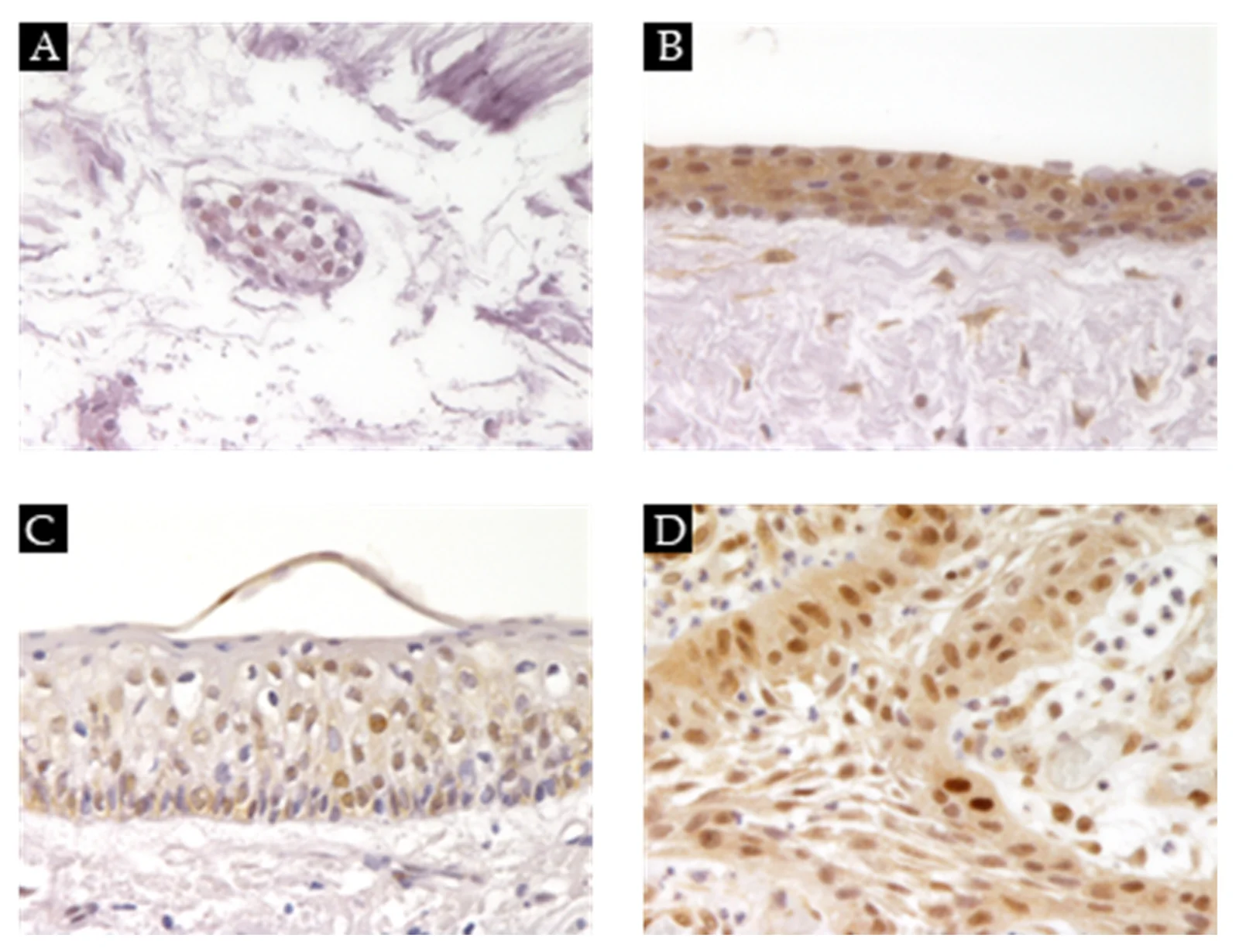
Detection of γH2AX expression by IHC. γH2AX expression was detected in DF (**A**), DC (**B**), OKC (**C**), and AM (**D**). (Original magnification: (**A**–**D**) ×400).

We detected γH2AX in the nucleus and cytoplasm in DF, DC, OKC, and AM (Figure 4). Nuclear staining or nuclear and cytoplasmic staining was defined as positive, whereas cytoplasmic staining alone was defined as negative. The positive rate of γH2AX was 15.6% in DF, 31.1% in DC, 29.1% in OKC, and 60.7% in AM. The positive rate of γH2AX expression was significantly higher in AM than in the other lesions by the χ^2^ test (Table 3).

**Figure 5:**
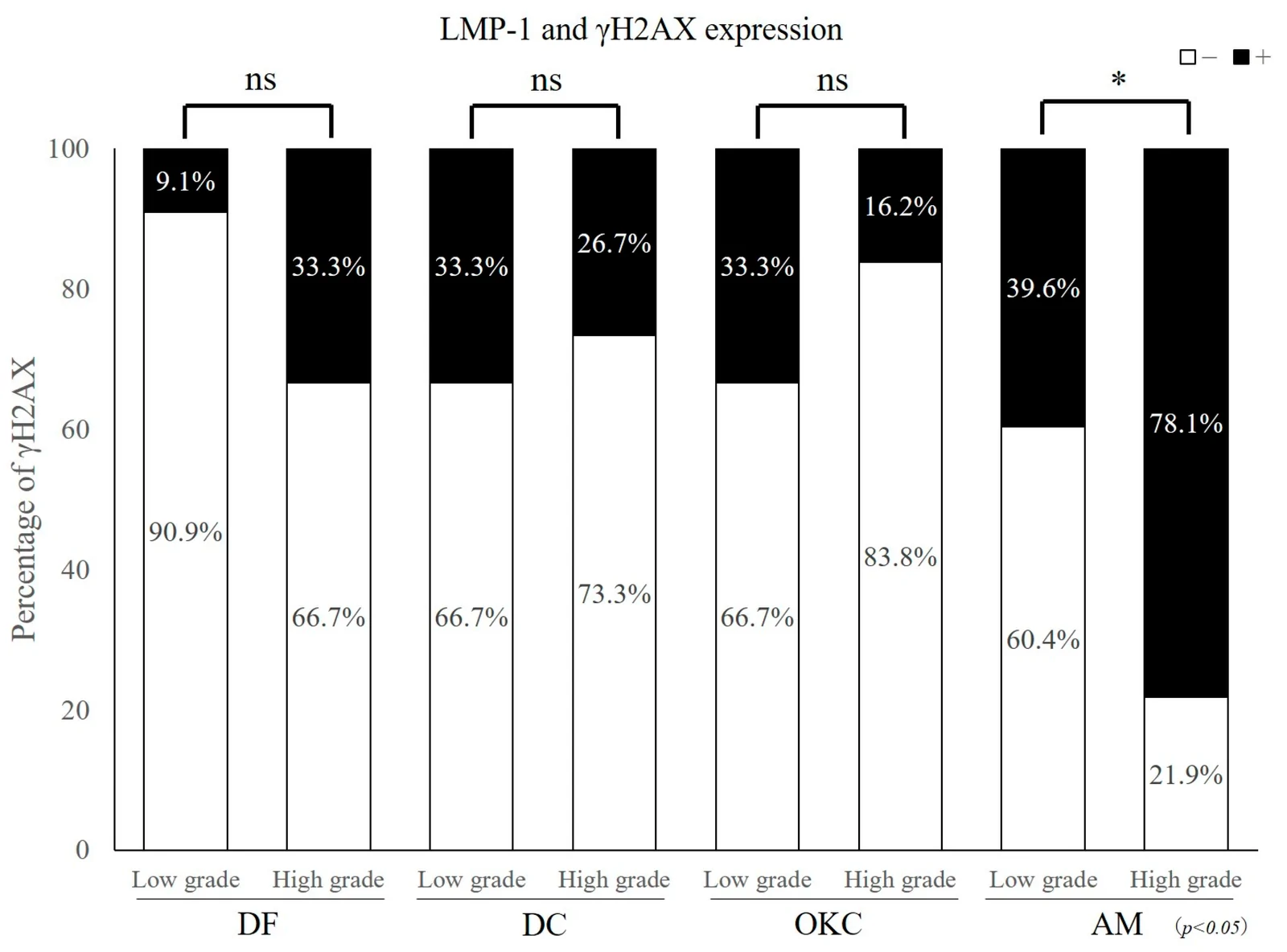
LMP-1 intensity and γH2AX expression. The positive rate of γH2AX in LMP-1 HG cases (78.1%) was significantly greater than in LG cases (39.6%) in AM. *p*-values were examined by Fisher’s exact test (* *p* < 0.05). ns: not significant.

In AM, the positive rate of γH2AX was 78.1% in HG cases and 39.6% in LMP-1 LG cases (Figure 5). The positive rate of γH2AX expression was significantly higher in LMP-1 HG cases than in LG cases (*p* < 0.05).

### 3.5. Relationship Between LMP-1 Expression Intensity and BRAF-V600E Mutant Protein in AM

**Figure 6:**
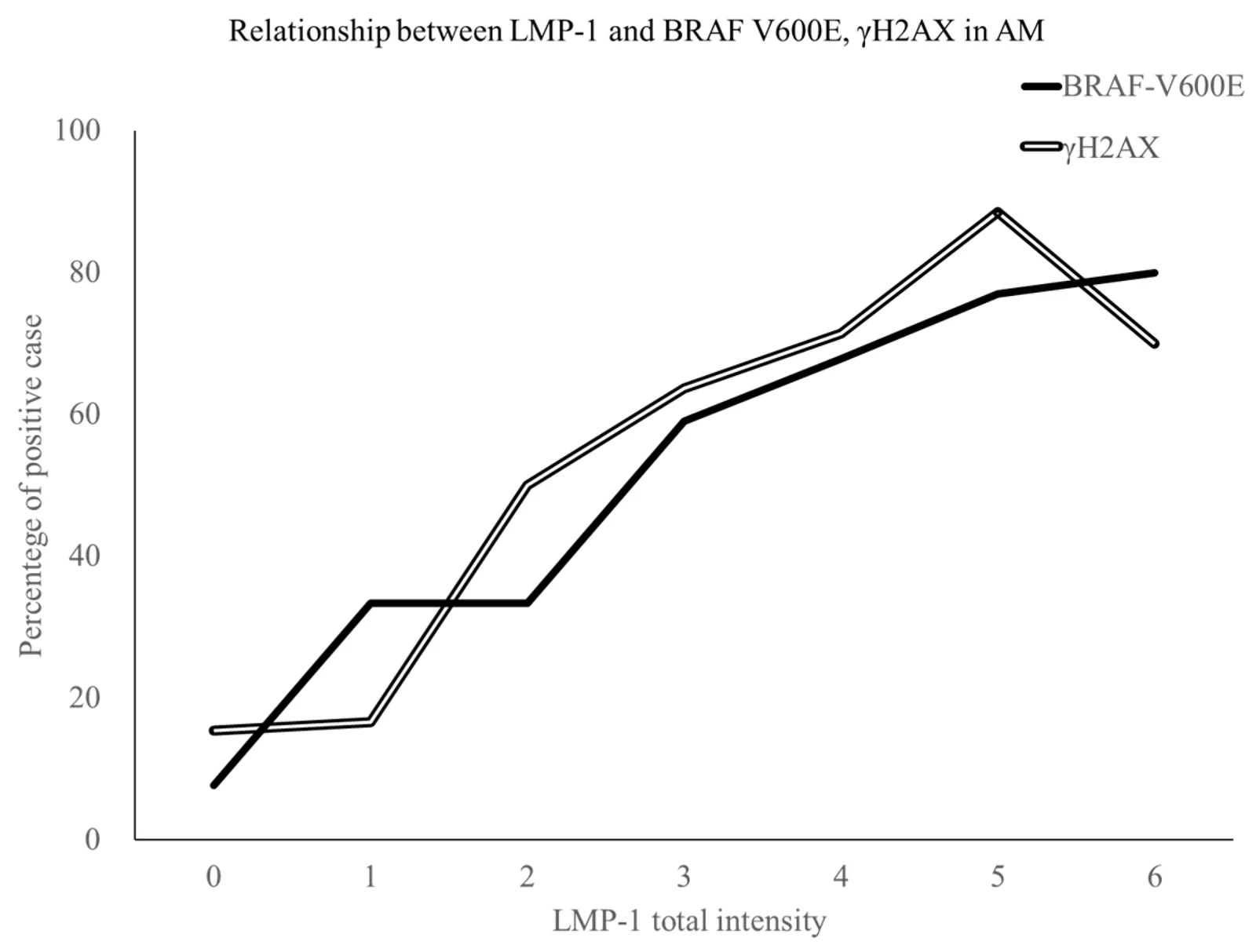
Relationship between LMP-1 and BRAF-V600E mutant protein, γH2AX in AM. The positive rate of BRAF-V600E mutant protein increased with increasing LMP-1 intensity (black line). The positive rate of γH2AX increased with increasing LMP-1 intensity (white lines).

We confirmed the positive rate of BRAF-V600E mutant protein and γH2AX classified by LMP-1 intensity in AM. The positive rates of BRAF-V600E mutant protein and γH2AX classified by LMP-1 intensity were 7.7% and 15.4% in intensity 0; 33.3% and 16.7% in intensity 1; 33.3% and 50.0% in intensity 2; 59.1% and 63.6% in intensity 3; 67.9% and 71.4% in intensity 4; 76.9% and 88.5% in intensity 5; and 80.0% and 70.0% in intensity 6. The positive rate of BRAF-V600E mutant protein and γH2AX increased with increasing LMP-1 staining intensity (Figure 6). We determined the relationships using binary logistic regression. Univariable binary logistic regression analysis showed that total intensity was significantly associated with the BRAF-V600E mutant protein and γH2AX positivity (BRAF-V600E mutant protein: OR = 1.78, 95% CI = 1.38–2.29, *p* < 0.001, γH2AX: OR = 1.87, 95% CI = 1.44–2.43, *p* < 0.001).

## 4. Discussion

We investigated the relationship between LMP-1 and BRAF-V600E mutant protein and γH2AX in odontogenic lesions using FFPE samples by IHC.

EphA2 expression in odontogenic epithelium and dental papilla has been reported [23]. EphA2 expression is confirmed in epithelial cells of normal oral mucosa [24]. The high proportion of EphA2 expression confirmed may not be an essential reason for odontogenic lesions because EphA2 is expressed in odontogenic epithelium and normal oral mucosa. There are few reports that focus on Eph receptors in odontogenic lesions, so the role of EphA2 in odontogenic lesions is unclear, but we confirmed EphA2 expression in the epithelium of DF, DC, OKC, and AM in this study.

Then, we detected the presence of BRAF-V600E mutant protein expression in each lesion by IHC. BRAF-V600E mutant protein expression was seen in 54.7% of AM cases, consistent with the previous reports [20,25]. It is reported that the MAPK pathway is involved in tooth formation [26]. Regarding the association between LMP-1 expression intensity and BRAF-V600E mutant protein expression, the proportion of BRAF-V600E mutant protein expression in AM was significantly greater in HG cases than in LG cases, but there was no significant difference among DF, DC, and OKC. Furthermore, since the positive rate of BRAF-V600E mutant protein increased with increasing LMP-1 total intensity in AM, LMP-1 and BRAF-V600E mutant protein expression may be related to AM.

Finally, we detected γH2AX expression in each lesion by IHC. γH2AX expression was mainly observed in the nucleus, but the cytoplasm was also stained in some cases. In AM, the positive rate of γH2AX expression was significantly higher in cases with HG LMP-1 expression than in LG cases. Zhang et al. showed that γH2AX expression was significantly higher in EBV-positive than in EBV-negative nasopharyngeal carcinoma, and that infection of an EBV-negative nasopharyngeal carcinoma cell line with EBV increased the expression of γH2AX [27]. Gruhne et al. reported that LMP-1 promoted genomic instability, increased γH2AX expression, and inhibited DNA repair [28]. Zhang et al. showed that γ-H2AX was detected in both the nucleus and cytoplasm following EV A71 infection [29]. In this study, since γH2AX expression in LMP-1 HG cases in AM was significantly higher than in LG cases and γH2AX expression increased with increasing LMP-1 total intensity in AM, EBV may cause DNA damage and inhibit DNA repair in AM.

In this study, we investigated LMP-1 and BRAF-V600E mutant protein expression in FFPE samples by IHC. IHC has its limitations in identifying molecular alterations, and it remains unclear whether LMP-1 high expression is involved in AM, whether BRAF-V600E or the microenvironment in AM causes LMP-1 high expression. Whether EBV is associated with the development of AM or with BRAF-related alterations remains unclear. Furthermore, because the present study was based exclusively on immunohistochemical analyses, EBV infection and BRAF mutation status were not independently confirmed by molecular analyses. Therefore, the observed findings should be interpreted as associations rather than evidence of a causal relationship. Therefore, further studies are needed to clarify the pathogenesis of EBV in odontogenic lesions.

## 5. Conclusions

BRAF-V600E mutant protein and γH2AX expression in AM were significantly higher in LMP-1 HG cases than in LG cases.

## Figures and Tables

**Table 1:** Clinical characteristics of DF, DC, OKC, and AM.

	DF (*N* = 45)	DC (*N* = 45)	OKC (*N* = 127)	AM (*N* = 117)
Age				
·Mean	14.3	43.7	40.7	40.0
·Range	(7~45)	(10~76)	(8~85)	(6~85)
Sex				
·Male	24	29	80	68
·Female	21	16	47	49
Location				
·Maxilla	28	9	41	11
·Mandible	17	36	86	106
Location				
·Anterior	19	5	23	28
·Posterior	26	40	104	89
Prognosis				
·Primary	45	45	125	111
·Recurrence	0	0	2	6

**Table 2:** Subtypes of AM.

Subtypes	*N*
Follicular	36
Plexiform	42
Mixed (Follicular, Plexiform)	20
Luminal	7
Desmoplastic	6
Acanthomatous	3
Granular cell	1
Mixed (Acanthomatous, Basal cell)	2

**Table 3:** Adjusted *p*-value for BRAF-V600E mutant protein and γH2AX.

	BRAF-V600E	γH2AX
DF vs. DC	2.8770	0.7598
DF vs. OKC	5.5619	0.7305
DF vs. AM	<0.0001	0.0013
DC vs. OKC	2.4969	5.0052
DC vs. AM	<0.0005	0.1196
OKC vs. AM	<0.0001	0.0013

## Data Availability

The data that support the findings of this study are available from the corresponding author upon reasonable request.

## Abbreviations

The following abbreviations are used in this manuscript:

EBV	Epstein–Barr virus
LMP-1	EBV-latent membrane protein-1
MPK	Mitogen-activated protein kinase
EphA2	Ephrin type-A receptor 2
HG	High grade
LG	Low grade
AM	Ameloblastoma
OKC	Odontogenic keratocyst
DC	Dentigerous cyst
DF	Dental follicle
FFPE	Formalin-fixed, paraffin-embedded
IHC	Immunohistochemistry
